# Effect of Micelle-Incorporated Cisplatin With Sizes Ranging From 8 to 40 nm for the Therapy of Lewis Lung Carcinoma

**DOI:** 10.3389/fphar.2021.632877

**Published:** 2021-03-08

**Authors:** Zhicheng Wang, Yumin Li, Tong Zhang, Hongxia Li, Zhao Yang, Cheng Wang

**Affiliations:** ^1^ Key Laboratory of Marine Drugs, Chinese Ministry of Education, School of Medicine and Pharmacy, Ocean University of China, Qingdao, China; ^2^ Qingdao Institute for Food and Drug Control, Qingdao, China; ^3^ Laboratory for Marine Drugs and Bioproducts of Qingdao National Laboratory for Marine Science and Technology, Qingdao, China

**Keywords:** cisplatin, nanomedicine, block copolymers, nanoparticles, lung cancer, drug delivery

## Abstract

Insufficient transport of therapeutic cargo into tumor bed is a bottleneck in cancer nanomedicine. Block copolymers are promising carriers with smaller particle size by ratio modification. Here, we constructed cisplatin nanoparticles with sizes ranging from 8 to 40 nm to study the permeability and therapy of Lewis lung carcinoma. We synthesized methoxypoly(ethylene glycol)_2000_-block poly(L-glutamic acid sodium salt)_1979_ loading cisplatin through complexation reaction. The cisplatin nanomedicine has high drug loading and encapsulation efficiency. *In vitro* data demonstrated that cisplatin nanoparticles had equivalent growth-inhibiting effects on Lewis lung carcinoma cells compared to free cisplatin. *In vivo* evidences showed cisplatin nanoparticles had superior antitumor effects on the Lewis lung carcinoma mouse model with no obvious side effects. All results indicated that optimizing the ratio of block copolymers to obtain smaller sized nanomedicine could act as a promising strategy for overcoming the inadequate accumulation in poorly vascularized tumors.

## Introduction

Nonspecific distribution and inadequate accumulation of therapeutic cargo are the main challenges in nanomedicine (Poon et al., 2020). Wilhelm et al. reported that conventional nanoparticles, only 0.7% injected dose, could accumulate in the tumor and that exploiting new strategies for overcoming the poor delivery efficiency is a formidable challenge to drug developers (Wilhelm et al., 2016).

**FIGURE 1:**
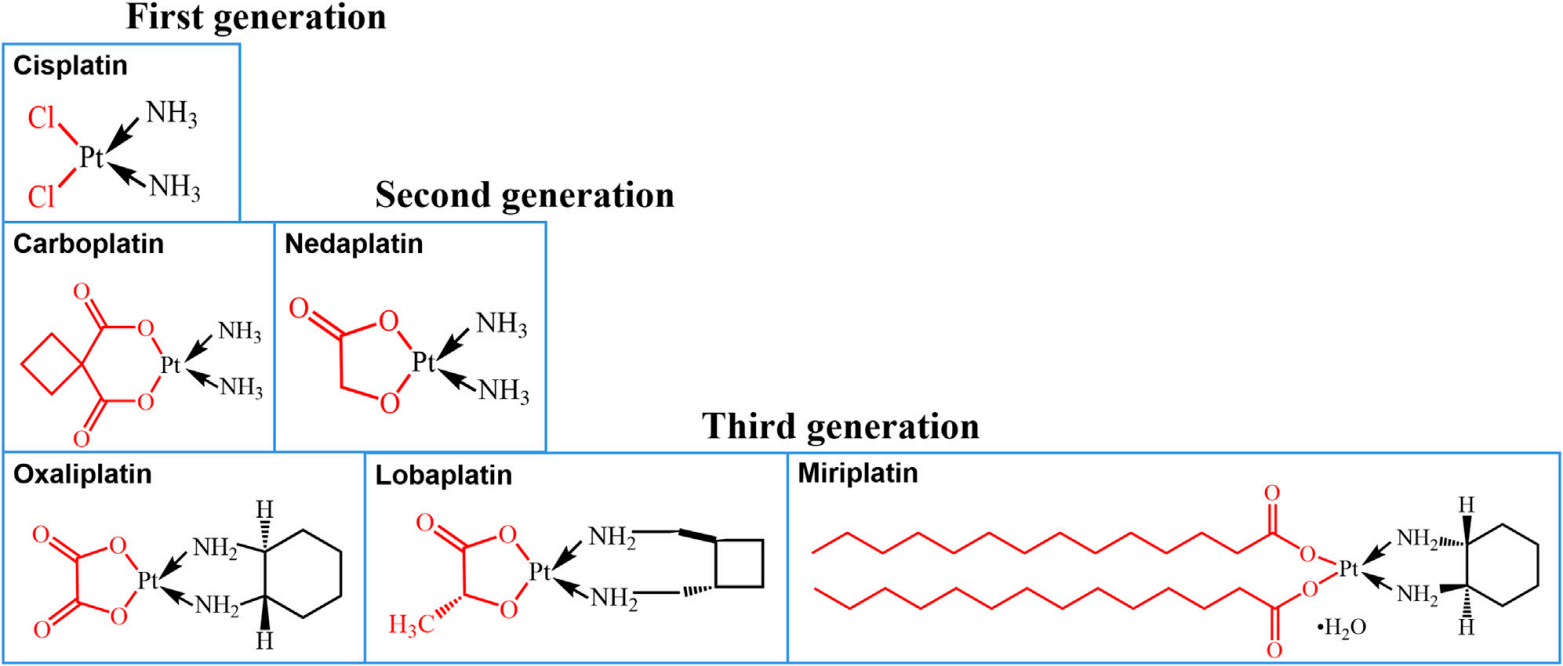
Platinum-based chemotherapy drugs with different chemical structures.

Lung cancer is difficult to be cured due to its poor penetration ascribing to its complex microenvironment (Gao et al., 2014; Bussard et al., 2016; Pautu et al., 2017; Shen et al., 2018). Cisplatin (CISP, Figure 1, trade name is Nuoxin^®^), a first-line chemotherapy agent for lung cancer since its launch in 1978 (Guo et al., 2018; Wiltshaw, 1979), causes interstrand and intrastrand crosslinking of nuclear DNA, leading to DNA damage and cell apoptosis (Xu et al., 2019). However, the high activity of the chloride ion as the leaving group leads to considerable nephrotoxicity and neurotoxicity, as well as other adverse reactions, resulting in limited clinical application (Ghosh, 2019). For this reason, in the subsequent design of platinum-based drugs, the chloride ion was replaced with the relatively stable cyclobutanecarboxylic acid and glycolic acid, respectively, to derive carboplatin (Calvert et al., 1982) and nedaplatin (Kuwahara et al., 2010). However, the commercial introduction of next-generation platinum drugs has been followed by a new challenge of high levels of cross-resistance between platinum agents. Further research revealed that the primary reason for the high degree of cross-resistance was that the platinum ion was liganded on the same side as the amino group. Consequently, in the tertiary development of platinum-based drugs, oxaliplatin (Wheate et al., 2010) and miriplatin (Liu et al., 2015) were generated by replacing the amino group with cyclohexane, and cyclobutane modification gave rise to lobaplatin (Martinez-Balibrea et al., 2015), whereas the more stable oxalic, lactic, and myristic acids substitute for the leaving group (Figure 1). Although the deleterious effects of the structurally modified platinum drugs were reduced, the antitumor spectrum of the drugs was narrowed and their efficacy diminished (Ghosh, 2019). As a result, CISP remains among the most effective chemotherapeutic agents used alone or in combination for the treatment of many neoplasms (Ghosh, 2019).

In addition, the particles smaller than 4–6 nm are filtered out of the blood and eventually passed in the urine (Dai et al., 2017), so only larger particles could escape kidney clearance. Kataoka and coworkers reported that particles below 30 nm have a superior penetration into poorly vascularized tumors (Cabral et al., 2011). Chauhan et al. also demonstrated that nanoparticles with diameters 12 nm are of ideal sizes for deeply penetrating tumor (Chauhan et al., 2012). NC-6004 was prepared through complexation of ionized carboxylic group of the polymer with CISP, and the efficacy of NC-6004 is being assessed in Phase III of the clinical trial in the United States (Plummer et al., 2011; Varela-Moreira et al., 2017). However, 80% of the nanoparticles formed from the hydrophilic fragments of methoxypoly(ethylene glycol)_12000_ and hydrophobic fragment of poly(L-glutamic acid sodium salt)_6000_ of the carrier are larger than 20 nm, and the particle size is not optimal (Nishiyama et al., 2003). Furthermore, methoxy-PEG is synthesized by ring opening polymerization of ethylene oxide using methanol as initiator. The polydispersity of MPEG < 5,000 Da can reach 1.01, which can ensure the MPEG derivative has lower polydispersity. Besides, its body clearance depends upon its molecular weight. The higher the molecular weight of PEG becomes, the slower it is cleared, and the liver clearance becomes more dominant (Pasut and Veronese, 2007). Of utmost importance is the molar mass of PEG affecting the composition of the proteins coating the nanoparticle surface (Schöttler et al., 2016), and the presence of distinct protein will influence the cellular uptake (Poon et al., 2020). In light of above considerations, it is essential to optimize the carrier to construct optimal cisplatin nanomedicine.

In this research, we successfully synthesized methoxypoly(ethylene glycol)_2000_–poly(L–glutamic acid sodium salt)_1979_ [MPEG_2000_-P(Glu)_1979_] block copolymers. The molecular weight ratio of MPEG to P(Glu) was according to the carrier of methoxypoly(ethylene glycol)_2000_-poly(DL-lactide)_1750_ (MPEG_2000_-PDLLA_1750_), which has been applied in the commercial production of Genexol-PM. Schöttler et al. also demonstrated that PEG2000 has a better effect of inhibiting the protein adsorption on particle compared to PEG_6000_ (Schöttler et al., 2016). To sum up, we have the following hypothesis that cisplatin nanoparticles (CISP-NPs) composed of MPEG_2000_-P(Glu)_1979_ should have smaller particle size with 8–40 nm and excellent tumor penetration leading to superior antitumor effect. To test the hypothesis, CISP-NPs were developed and characterized *in vitro*. Furthermore, we preliminarily evaluate its antitumor efficacy in a xenograft mouse model of Lewis lung carcinoma to provide more evidence toward new drug registration. Finally, the results confirmed that the strategy is applicable.

## Materials and Methods

### Materials

Methoxypoly(ethylene glycol) (MPEG, Mn = 2000, Aldrich, United States), γ-benzyl-L-glutamate-N-carboxy anhydride (BLG-NCA), dichloromethane, pyridine, *P*-toluene sulfonyl chloride (TsCl), absolute ether, ammonium hydroxide, benzene, dimethylsulfoxide (DMSO) and 1,4-dioxane were purchased from Sinopharm Chemical Reagent Co., Ltd. (Shanghai, China). Petroleum ether was obtained from Fuyu Chemical Corp. (Shanghai, China). Simethicone was purchased from Yongda Chemical Corp. (Tianjin, China). Sodium bicarbonate was purchased from Dingshengxin Chemical Corp. (Tianjin, China). Tetrahydrofuran and sodium hydrate were purchased from Huirui Chemicals Corp. (Tianjin, China) and Jinshan Pharmaceutical Corp. (Sichuan, China), respectively. Spectrographic grade potassium bromide (KBr) was obtained from Sinopharm Chemical Reagent Co., Ltd. (Shanghai, China). HPLC-grade acetonitrile was purchased from Anaqua Chemical Supply (Houston, TX, United States). *Cis*-platinum was purchased from Meryer (Shanghai, China).

Dulbecco’s Modified Eagle’s Medium (DMEM) High Glucose and fetal bovine serum (FBS) were purchased from Gibco (Shanghai, China). Penicillin/streptomycin and 0.25% (w/v) trypsin-0.1% (w/v) Ethylenediaminetetraacetic acid (EDTA) were bought from Solarbio (Beijing, China). 3-(4,5-Dimethyl-thiazol-2-yl)-2,5-diphenyl-tetrazolium bromide (MTT) was purchased from Sigma (Shanghai, China). Culture flasks and dishes were obtained from Corning (NY, United States).

Lewis lung carcinoma (LLC) cell line, derived from the C57BL/6 mouse, was purchased from the Type Culture Collection of the Chinese Academy of Sciences (Shanghai, China). LLC cells were maintained in DMEM with 10% FBS in the humidified atmosphere containing 5% CO_2_ at 37°C.

Healthy, six-to-eight-week-old male C57BL/6 mice were purchased from Qingdao Daren Fortune Animal Technology Co., Ltd. (Qingdao, China). Food and water were given to all mice *ad libitum*. Feeding temperature was controlled at 20–22°C, relative humidity 50–60%, light and dark cycles for 12 h. All experiments were conducted in accordance with the guidelines of the Institutional Animal Care and Use Committee, Ocean University of China.

### Synthesis of MPEG-P(Glu) Block Copolymer

**FIGURE 2:**
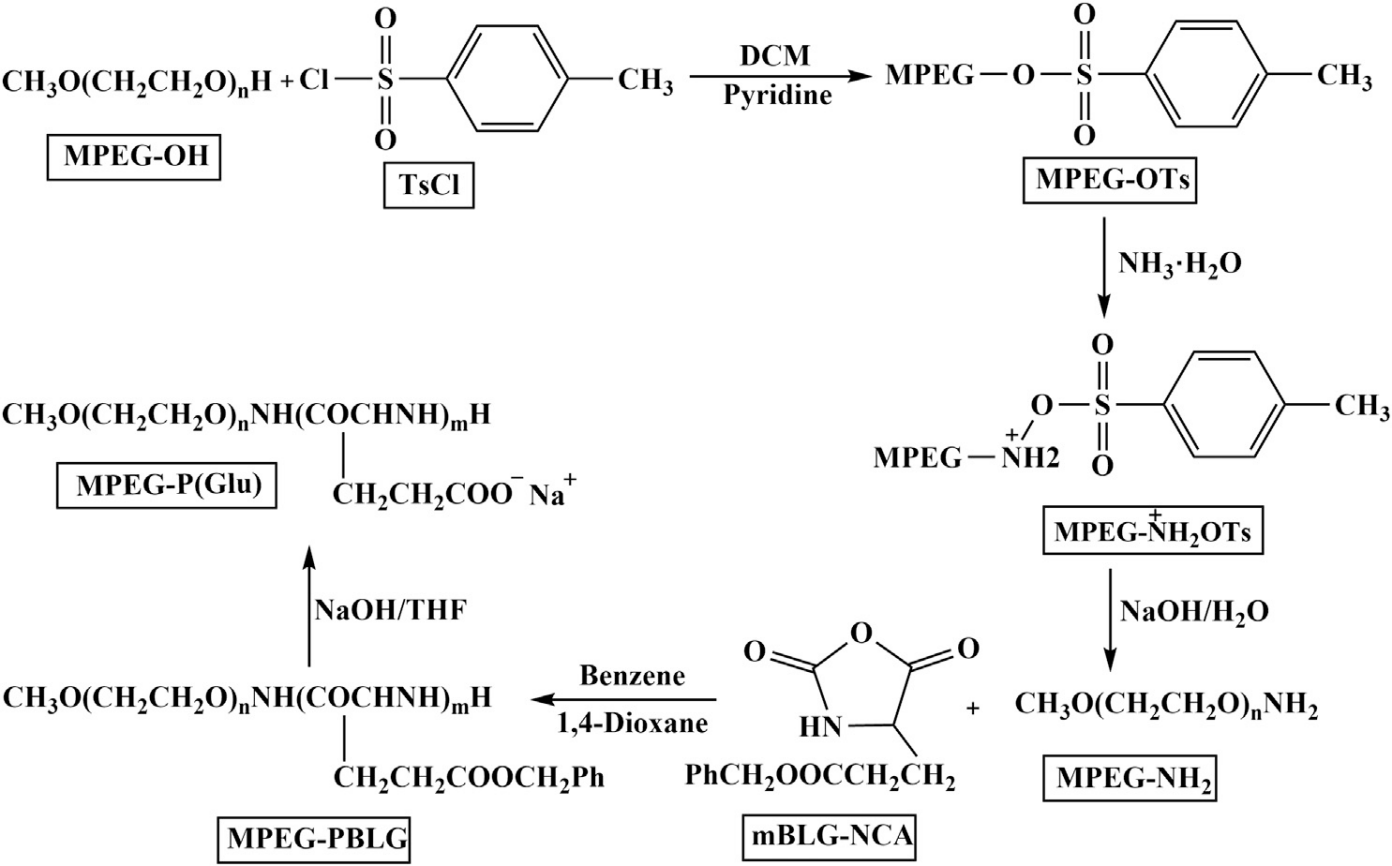
The route of synthesis sodium salt of MPEG-P(Glu).

MPEG-P(Glu) block copolymer was synthesized as previously reported (Cabral et al., 2007) (Figure 2). First, methoxypoly(ethylene glycol) amine (MPEG_2000_-NH_2_) was prepared by the method of toluene sulfonate esterification. MPEG_2000_ (40 g, 20 mmol) and CH_2_Cl_2_ (200 ml) were added in a 3-neck round-bottomed flask (500 ml), stirred for 5 min. Subsequently, pyridine (150 ml, 1.89 mol) and TsCl (7.6 g, 40 mmol) were added into the solution above and stirred for 24 h under nitrogen protection at 30°C and then cooled to 0°C. The reaction mixture was poured into hydrochloric acid solution stirred for 15 min and the CH_2_Cl_2_ layer was collected. The aqueous phase was extracted from 200 ml dichloromethane (three times). The combined organic layer was washed with brine, aqueous sodium bicarbonate, and brine successively, dried over anhydrous sodium sulfate overnight. Next, tetrahydrofuran was added and stirred for 5 min. Then, the acquired white powder (MPEG_2000_-OTs) was washed 2–3 times with diethyl ether and dried under vacuum at 25°C for 4 h. After MPEG_2000_-OTs (7 g, 3.25 mmol) was dissolved in aqueous ammonia and stirred for 8.5 h at 135°C, MPEG_2000_-NH_2_ was obtained by isolation and purification, dried for 4 h. The following steps were applied to generate methoxypoly(ethylene glycol)-poly (γ-benzyl-L-glutamate) (MPEG-PBLG) block copolymer. MPEG-PBLG was synthesized by ring-opening polymerization of BLG-NCA monomer using MPEG_2000_-NH_2_ as macro initiator, and then the benzyl group was deprotected following the literature procedure (Song et al., 2012). BLG-NCA (4 g, 15.2 mmol) was suspended in a mixed solution of benzene (64 ml) and dioxane (16 ml) and then MPEG_2000_-NH_2_ (1.2 g, 0.6 mmol) was added. The reaction mixture was reacted under nitrogen protection at ambient temperature for 4 days. After the reaction, the mixture was poured into a large volume of anhydrous ethanol and stirred for 1 h, rested for 30 min, suction filtration, and washed with little ethanol. The precipitated product was dried under vacuum at 30°C for 12 h. At last, the benzyl group was removed from protection by blending with 0.025 N NaOH at ambient temperature to produce MPEG-P(Glu).

### Characterization of MPEG–NH_2_, MPEG-PBLG, and MPEG-P(Glu)

The structure of MPEG-OH, MPEG–NH_2_, and MPEG-PBLG was confirmed by Fourier-transform infrared (FT-IR) analyzer (Nexus 470 by Nicolet Instruments America). 2% (w/w) of the sample was dispersed in KBr and then compressed it into slices. Each slice was scanned between 400 and 4,000 cm^−1^.

The structure and purity of MPEG_2000_-PBLG and MPEG_2000_-P(Glu) were determined by ^1^H nuclear magnetic resonance spectroscopy (^1^H-NMR) instrument (JNM-ECP600, JEOL TMS, Tokyo, Japan) at 25°C. MPEG_2000_-PBLG and MPEG_2000_-P(Glu) were dissolved in deuterated dimethyl sulfoxide (DMSO-d_6_, 500 MHz), D_2_O to a concentration of approximately 10 mg/ml, respectively.

### Preparation and Characterization of CISP-NPs

#### Preparation of CISP-NPs

**FIGURE 3:**
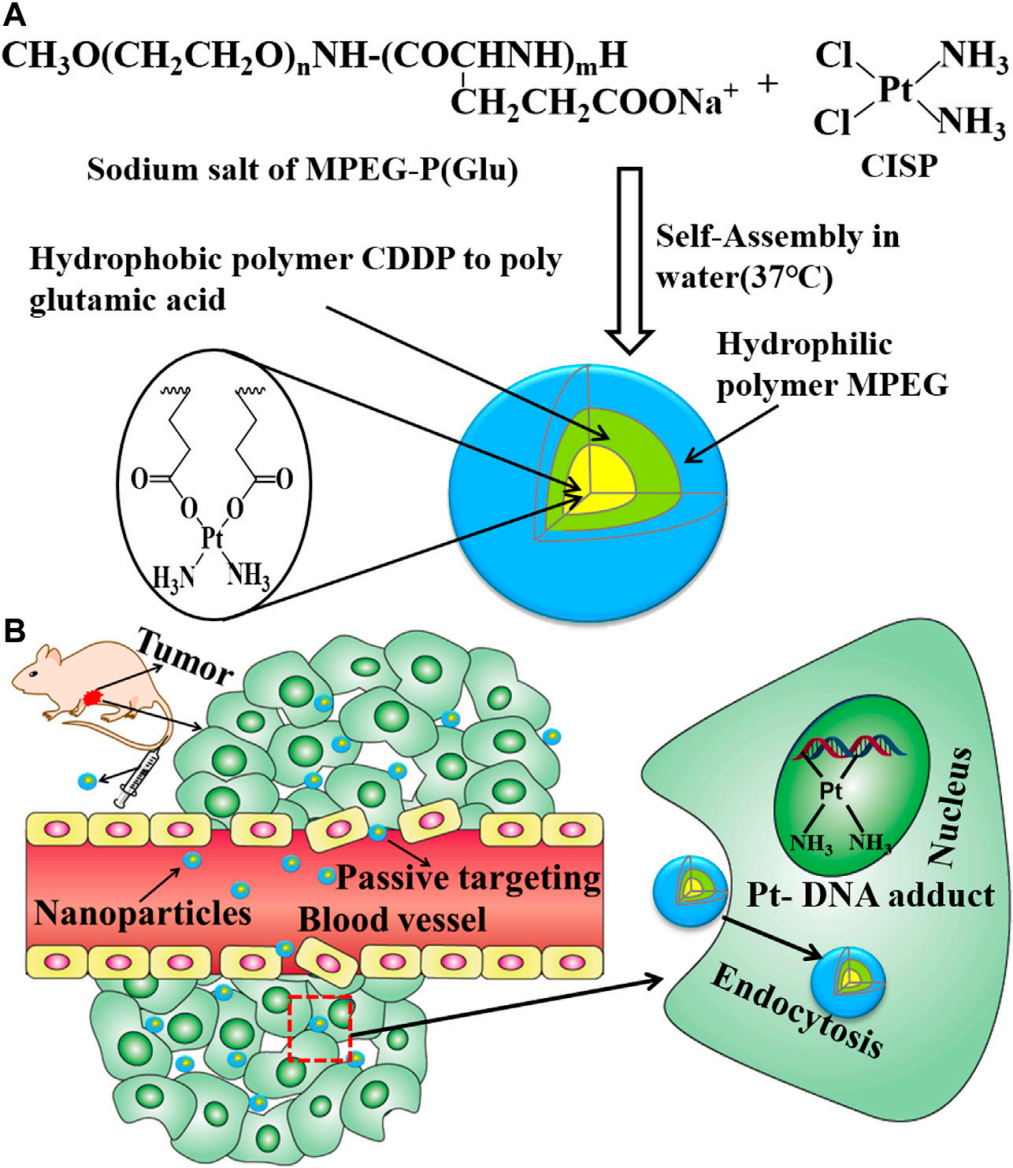
**(A)** Preparative procedure of CISP-NPS. **(B)** The intracorporal process of CISP-NPs after administration.

CISP-NPs were prepared according to the procedure described previously with slight modifications (Nishiyama et al., 2003; Uchino et al., 2005). Figure 3A was the preparation scheme of CISP-NPs. MPEG_2000_-P(Glu)_1979_ and CISP (CISP)/(Glu) = 1.0) were dissolved in distilled water and shaken at 37°C for 72 h in the dark until the mixture was dissolved completely. The hydrophilic chain of the block copolymer, MPEG, made up the outer shell of the micelles. Poly(glutamic acid) and CISP were hydrophobic chains, a polymer-metal complex-forming chain, which formed the inner core of the micelles. Once the micelle was formed, free CISP was eliminated by dialysis with a membrane of the molecular cut-off of 1 kDa. The water was freshened every 2 h during the previous 8 h. After dialysis for 12 h, the micelle solution was filtered with 0.22 μm filters (Millipore).

#### Characterization of CISP-NPs

The morphological characteristics of CISP-NPs were investigated by transmission electron microscope (TEM, EOL JEM-1200EX, Ltd., Japan) (Weng et al., 2017). Briefly, the micelle solution was dropped onto a 200-mesh copper grid coated with a Formvar-carbon support film and stained with 2% phosphotungstic acid. Extra liquid was wiped with filter paper and then slowly dried at room temperature prior to measurement. Moreover, the mean diameter of the CISP-NPs was measured by Malvern Zetasizer Nano ZS90 (Malvern, Worcestershire, United Kingdom) particle size analyzer, as well as polydispersity index (PDI). Acquisition of three-dimensional images of the sample used atomic force microscopy (AFM, Agilent) (Zhu et al., 2013). Briefly, a droplet of 0.5 mg/ml polymer samples was spread on the polyethyleneimine-coated glass cap and dried naturally. Scanning was done with a probe tip, then the sample was imaged with Nano-scope III (Digital Instrument VEECO).

To identify whether CISP was encapsulated in MPEG-P(Glu), samples analyzed by the powder X-ray diffractometer (XRD, Bruker D8 ADVANCE) of which the voltage and current employed were 40 kV and 30 mA, respectively (Pansini et al., 2017). X-ray diffraction (XRD) patterns of MPEG-P(Glu), CISP, and CISP-NPs lyophilized powder and physical mixture of CISP and MPEG-P(Glu) (30:70) were obtained from 5° to 60° at a 0.02° step and 5°/min scan speed.

### Drug Loading and Encapsulation Efficiency of CISP-NPs

Drug loading (DL) and encapsulation efficiency (EE) of the CISP-NPs were determined as follows: briefly, lyophilized CISP-NPS was dissolved in double distilled water and then hydrochloric acid (2 mol/L) was used to disrupt the structure of micelles. Afterwards, the solution was filtered by 0.22 μm syringe filter. The subsequent filtrate was measured by high performance liquid chromatography (HPLC) and inductively coupled plasma atomic emission spectroscopy (ICP-AES), respectively. According to the following formulas, DL and EE of CISP-NPs were calculated:

$$\mathrm{DL=}\frac{\mathrm{weight}\;\mathrm{of}\;\mathrm{CISP}\;\mathrm{in}\;\mathrm{micelle}}{\mathrm{weight}\;\mathrm{of}\;\mathrm{the}\;\mathrm{polymer}\;\mathrm{and}\;\mathrm{CISP}}\mathrm{\times 100\%},$$ (1)

$$\mathrm{EE=}\frac{\mathrm{weight}\;\mathrm{of}\;\mathrm{CISP}\;\mathrm{in}\;\mathrm{micelle}}{\mathrm{weight}\;\mathrm{of}\;\mathrm{the}\;\mathrm{feeding}\;\mathrm{CISP}}\mathrm{\times 100\%}.$$ (2)

Determination of CISP concentration by HPLC (Shimadzu, Kyoto, Japan) is performed with the LC-20AT solvent delivery unit, the SPD-20A UV/VIS detector, the plus manual sampler, and the HT-230A column heater (Tianjin Hengao Technology, Tianjin, China). The chromatographic analysis was conducted on a Hypersil NH_2_ (250 mm × 4.6 mm, 5μm, Elite, Dalian, China) with the detection wave length at 310 nm. The flow rate of mobile phase, ethyl acetate-methanol-dimethyl formamide-deionized water (25:16:5:5, v/v), was 0.8 ml/min whilst the column temperature was maintained at 25°C. The standard curve equation is A = 696.37*C-913.69 (A for the area of peak; C for the concentration of CISP-NPs) and the correlation coefficient is 0.9999.

Another test method, the metal ions standard solution, was the configuration at concentrations of 8 µg/L, 16 µg/L, 24 µg/L, 32 µg/L, and 48 µg/L to obtain standard curve. After the nanoparticles were nitrated with nitric acid (5 mg/ml), the manganese content of solution was measured with optima 8000 ICP-AES.

### Cytotoxicity Evaluation

Cytotoxicity of free CISP, CISP-NPs, and MPEG-P(Glu) polymer was determined by the MTT assay on LLC cells. LLC cells were seeded in 96-well culture plates (Jet Biofil) at a density of 6 × 10^3^ cells/well and incubated for 24 h. Cells were then treated with a series of drugs at different concentrations of free CISP (DMSO <0.5%), CISP-NPs, and MPEG-P(Glu) polymers for 24, 48, and 72 h, respectively. Tests were repeated for 6 times. Briefly, at the determined incubation-time, 20 μl of 5 mg/ml MTT was added per well of the plate. After incubation for another 4 h at 37°C, purple formazan crystals were observed. The supernatant was aspirated off gently, and 150 μl/well of DMSO was added into each well and the plate was gently shaken for 10 min at room temperature to dissolve the formazan crystals, and the absorbance of the resulting DMSO solution at 490 nm was measured with a SPECTRA-max M5 microplate reader (Molecular Devices, United States). Cells without drug treatment were served as a negative control with a 100% survival rate, while cells without MTT were used as a blank control to calibrate the spectrometer meter. Relative cell viability (%) in comparison to control cells was calculated as (Abs_sample_/Abs_control_) × 100%, expressed as the mean ± SD of six measures.

### Flow Cytometry Analysis for Cell Cycle

Cell cycle analysis was evaluated as described below (Wang et al., 2013). In brief, LLC cells were seeded at a density of 5 × 10^5^ cells in each well in a six-well plate (NEST Biotechnology) followed by incubation for 12 h. Then, culture media was substituted with fresh medium containing free CISP or CISP-NPs formulations at dosage of 12.55 μg/ml of CISP and cultivated for 48 h, with untreated cells as the negative control. At the end of the incubation, the collected cells (1 × 10^6^) were centrifuged at 1,000 rpm for 5 min, resuspended and washed twice with ice-cold PBS, and then fixed with 75% cold ethanol. After storage for 24 h at 4°C, the cell suspension was centrifuged again and washed twice with cold PBS and then incubated with the freshly prepared staining solution containing propidium iodide (PI) and RNase (Beyotime, Haimen, China) at 37°C under light-proof conditions for 30 min. Subsequently, the DNA content was determined by Cytomics FC500 MPL flow cytometry system (Beckman Coulter, United States). The percentage of cell populations in each stage of the cell cycle, including G0/G1, S, and G2/M phases, was assessed using MultiCycle AV software (San Diego, CA).

### Antitumor Activity

Antineoplastic activity of CISP-NPs was elucidated in the mouse xenograft model. 1 × 10^6^ LLC cells were inoculated subcutaneously into the right axillary space of the C57BL/6 mice. Briefly, the treatment was initiated when the tumor size reached an average of 7–9 mm and randomly divided into four groups (n = 6 for per group). Mice were separately injected with saline and MPEG-P (Glu) polymer, free CISP (6 mg/kg), CISP-NPs (6 mg/kg CISP or 12 mg/kg CISP) via the tail vein on days 7, 10, 13, 16, and 19, respectively. On day 21, three mice in each group were executed by cervical dislocation, followed by the tumor, heart, liver, spleen, and kidney which were excised with histopathological examination by Hematoxylin-Eosin (H&E) staining. The remaining three mice in each group were further used for survival experiments.

### Histopathology Evaluation

Histopathological impairment evaluated by hematoxylin and eosin (H&E) assay according to manufacturer’ s instruction. Liver, heart, spleen, and kidney from treated mice were stained with hematoxylin-eosin for assessment of toxicity. Briefly, tumor, heart, liver and spleen and kidney tissues in different groups were harvested and then fixed in 10% neutral buffered formalin. The fixed tissues were subsequently embedded in paraffin and sectioned in 3-μm increments. The sections were dewaxed by dipping them in xylene and then placed on glass slides and stained with H&E. Observations were made by optical microscopy (OLYMPUS BX60, Japan) with corresponding image analysis software (OLYMPUS DP25, Japan).

### Statistical Analysis

All statistical data were expressed as means ± standard deviation (SD) and total experiments were performed at least three replicates. The significant difference between the groups were examined by Student’s *t*-test. *p < 0.05, **p < 0.01, and ***p < 0.001 were deemed statistically significant.

## Results and Discussion

### Preparation and Characterization of MPEG-NH_2_, MPEG-PBLG, and MPEG-P(Glu)

**FIGURE 4:**
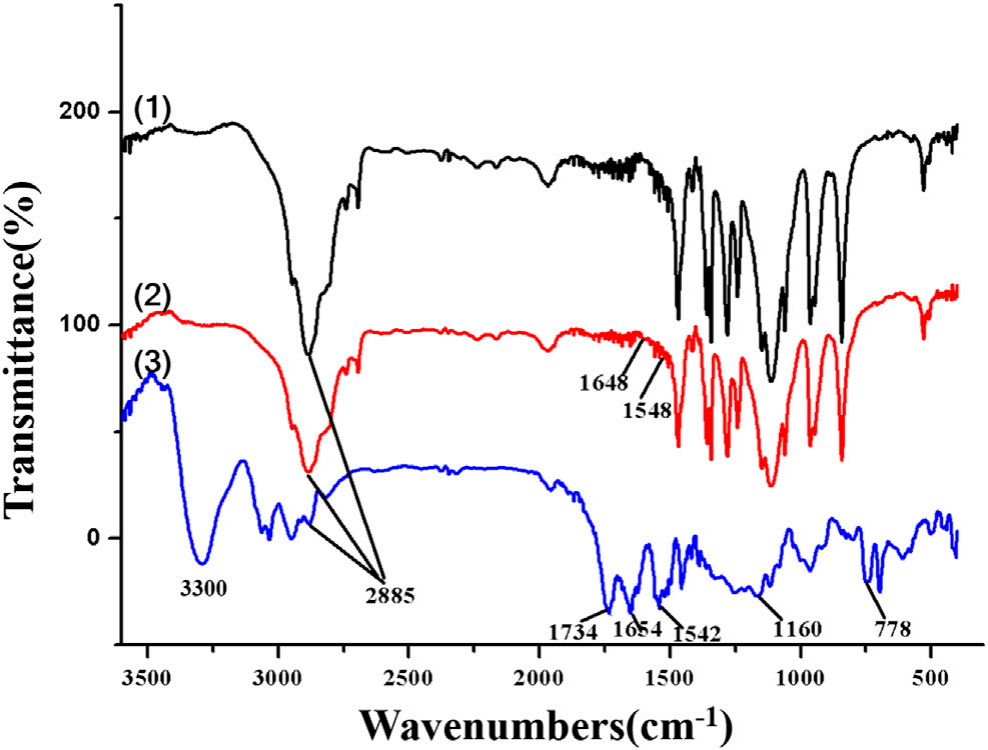
The FTIR spectra of (1) MPEG-OH, (2) MPEG-NH_2_, and (3) MPEG-PBLG.

**FIGURE 5:**
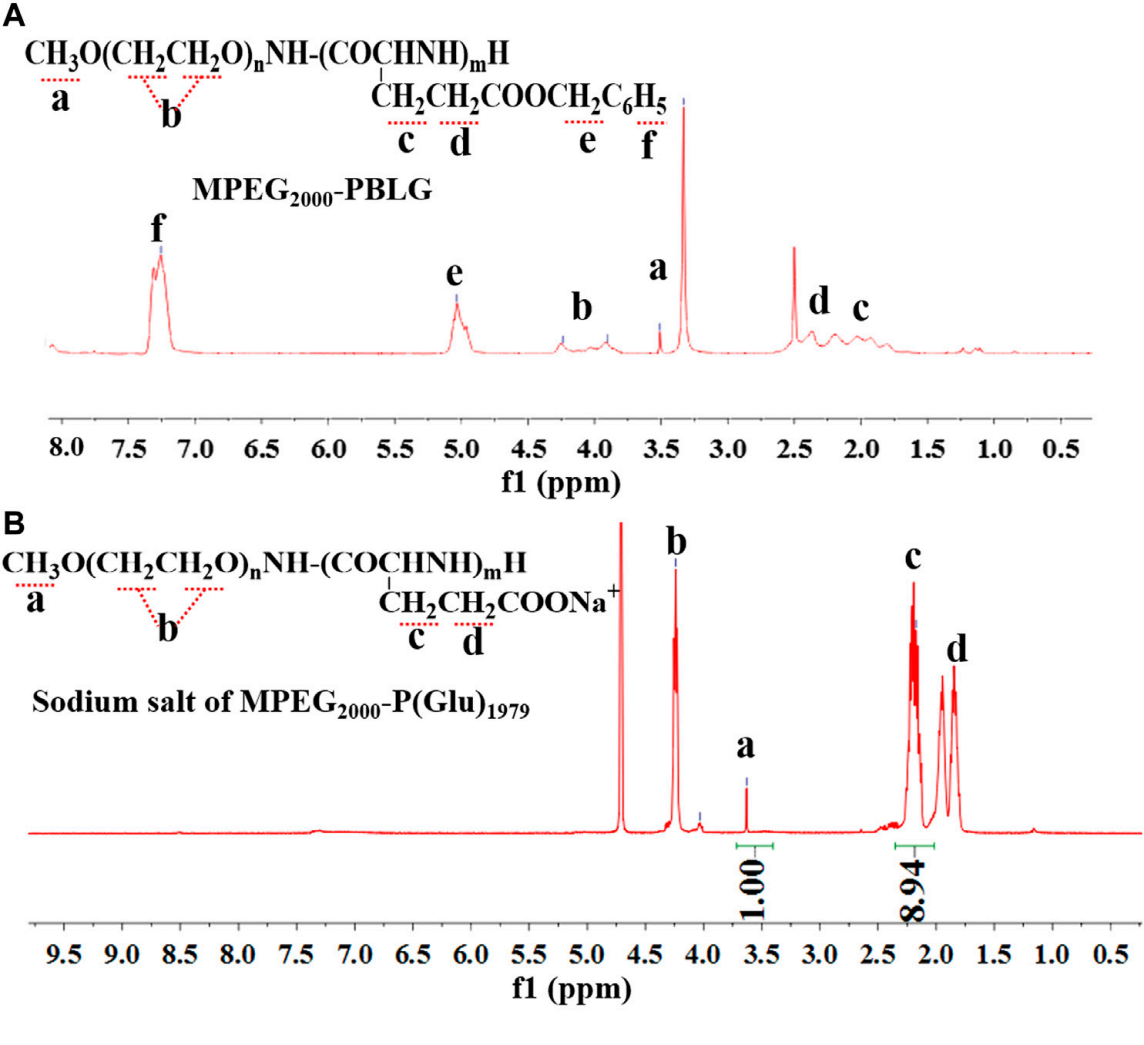
The ^1^H NMR spectra for MPEG_2000_-PBLG **(A)** and sodium salt of MPEG_2000_-P(Glu)_1979_ **(B)**.

The FTIR spectra of MPEG_2000_-OH, MPEG_2000_-NH_2_, and MPEG_2000_-PBLG were shown in Figure 4. The characteristic peak of amine bond appearing at 1,648 and 1,548 cm^−1^ indicated the N-H stretch of MPEG_2000_-NH_2_ [Figure 4(2)]. The characteristic IR absorption frequencies for MPEG_2000_-PBLG [Figure 4(3)] were 3,300 (OH, NH Str), 2,885 (CH_2_ Str), 1,734 (C = O Str), 1,654 and 1,542 (CONH Str), and 778 (CH bend aromatic). The ^1^H-NMR spectra (Figure 5) also showed that MPEG_2000_-PBLG and MPEG_2000_-P(Glu) block copolymers were successfully synthesized. In the ^1^H NMR spectrum (Figure 5A), five major signals were observed at 3.50 (Figure 5A-a), 3.90–4.4 (Figure 5A-b), 1.791–2.180 (Figure 5A-c), 2.364 (Figure 5A-d), 5.034 (Figure 5A-e), and 7.256 (Figure 5A-f) ppm, which represented the carbons of (CH_3_O−, 3H), (−CH_2_CH_2_O−, 4H), (−CH_2_CH_2_COO^−^Na^+^, 2H), (−CH_2_CH_2_COO^−^Na^+^, 2H), (C_6_H_5_CH_2_−, 2H), and (C_6_H_5_−, 5H), respectively. Taking above mentioned results into consideration, its proved MPEG_2000_-PBLG was successfully synthesized. The deprotection of the benzyl group was performed by mixing with 0.025 N NaOH at ambient temperature to obtain MPEG_2000_-P(Glu), the peak of which at δ 5.034 and 7.256 ppm disappeared, confirming the complete deprotection (Figure 5B). The peak of 3.630 (Figure 5B-a), 4.24 (Figure 5B-b), 2.17 (Figure 5B-c), and 1.85–1.95 (Figure 5B-d) ppm represented the carbons of (CH_3_O−, 3H), (−CH_2_CH_2_O−, 4H), (−CH_2_CH_2_COO−Na^+^, 2H), and (−CH_2_CH_2_COO^−^Na^+^, 2H), respectively. The whole average molecular weight of MPEG_2000_-P(Glu)_1979_ was 3,979 Da, while the number of units of the P(Glu) was 13.

### Characterization of CISP-NPs

**FIGURE 6:**
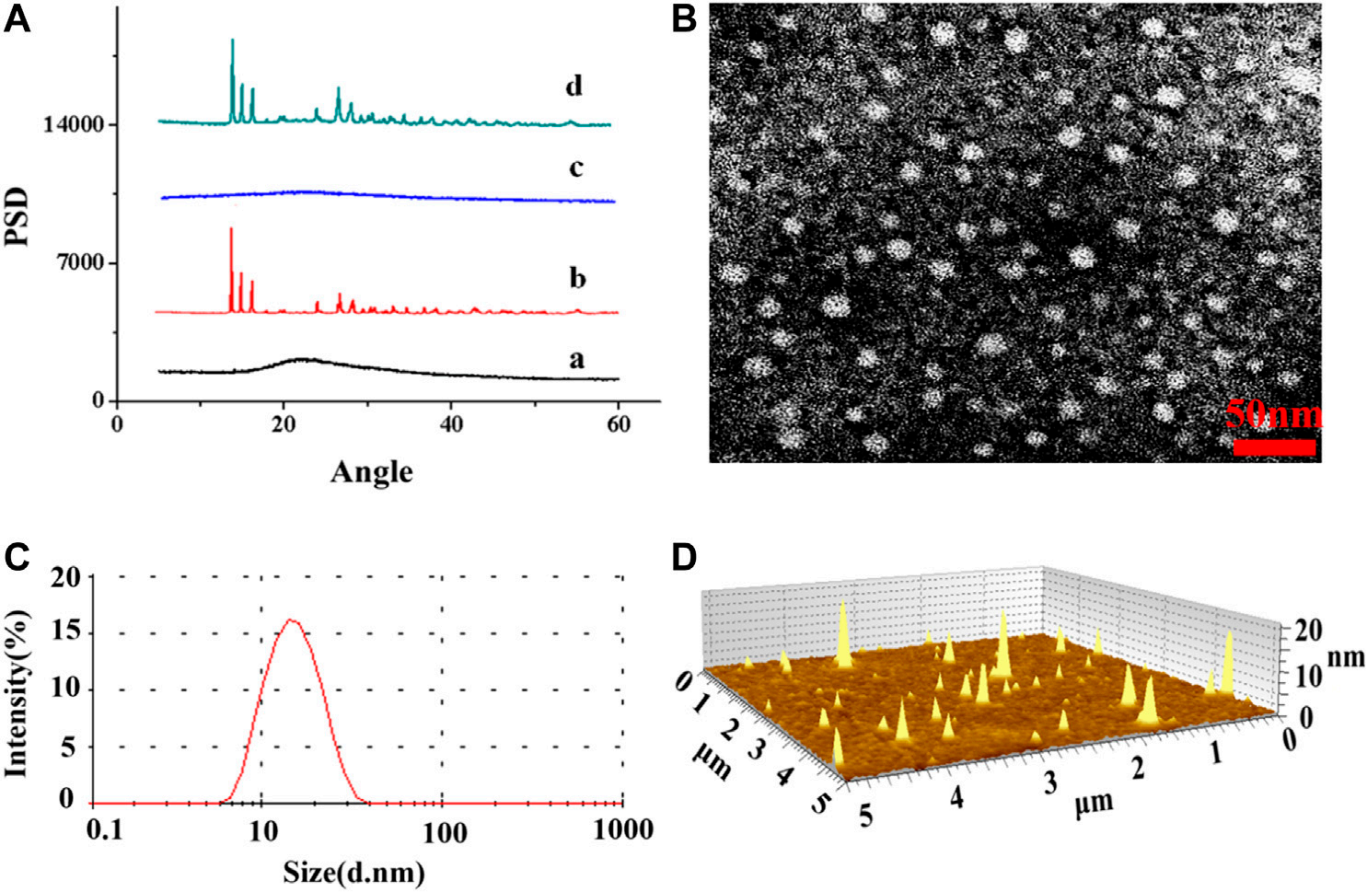
**(A)** XRD patterns spectra of **(A)** MPEG_2000_-P(Glu)_1979_ material, **(B)** CISP crystalline powder, **(C)** CISP-NPs lyophilized powder (DL = 31.7%), **(D)** physical mixtures of CISP and MPEG_2000_-P(Glu)_1979_ (30:70); **(B)** TEM image of CISP-NPs and the scale bar 50 nm; **(C)** particle size distribution of CISP-NPs; **(D)** AFM image of CISP-NPs.

As CISP is widely used for chemotherapy drug in clinical trials, it still has a lot of side effects, especially nephrotoxicity, which make many patients suffered. In order to resolve this problem, nanotechnology-based delivery systems were devised to be capable of loading all sorts of drugs in recent decades (Xu et al., 2017). In this study, the biocompatible methoxypoly(ethylene glycol)-*b*-poly(L-glutamic acid) (MPEG-P(Glu)) was used to encapsulate CISP. The typical XRD patterns of pure MPEG_2000_-P(Glu)_1979_ material (1), CISP crystalline powder (2), CISP-NPs lyophilized powder (DL = 31.7%) (3), and the mixture of CISP and MPEG_2000_-P(Glu)_1979_ (4) were illustrated in Figure 6A. The XRD spectrum of free CISP showed many sharp peaks confirming that CISP was a crystalline compound, where narrow peaks represented some paracrystalline phases. While blank carrier was amorphous, the mixture of CISP and MPEG_2000_-P(Glu)_1979_ presented some peaks (in the 2θ range of 10–30°) suggesting crystallizability of CISP. However, CISP-NPs lyophilized powder was not detected with crystalline peaks, and the crystalline diffraction peaks of CISP disappeared or were masked since the CISP was encapsulated into CISP-NPs in an amorphous form. Furthermore, it was reported that the change of XRD patterns was due to the possible inhibition of CISP crystallization by polymer matrices. Compared with free CISP, CISP-NPs showed superior dissolution rate and water solubility through transforming a crystalline drug into its amorphous form (Hancock and Zografi, 1997; Lobmann et al., 2011). The amorphous crystalline form of CISP inside the MPEG_2000_-P(Glu)_1797_ contributes to its sustained release (Ji et al., 2016). The drug-polymer amorphous system could reach the goal of improving solubility and bioavailability of poorly soluble drugs and the stability of the amorphous could be improved by distributing the drug in an amorphous state in highly dispersed carrier materials (Li and Huang, 2008).

The drug loading (DL) and encapsulated efficiency (EE) of CISP-NPs were (28.54, 27.82%) and (85.63, 83.74%), which were measured by HPLC and ICP-AES, respectively. Optimizing nanocarrier MPEG2000-P(Glu)1979 and the particle size of nanodrug, the average size of CISP-NPs obtained in this study was (18 ± 1.33) nm (Figure 6C), with PDI of 0.185 and zeta potential of −21 mV, which could enhance tumor accumulation and aggregation via the EPR effect and achieve longer blood circulation. The TEM (Figure 6B) and AFM (Figure 6D) image revealed that CISP-NPs was spherical in shape and uniformly distributed without significant agglomeration. The particle size of CISP-NPs ranged from 8 to −40 nm with an average size of 18 ± 1.33 nm, which not only could evade renal clearance but also advance the penetration of nanodrugs into tumor tissues.

### Cytotoxicity Assay

**FIGURE 7:**
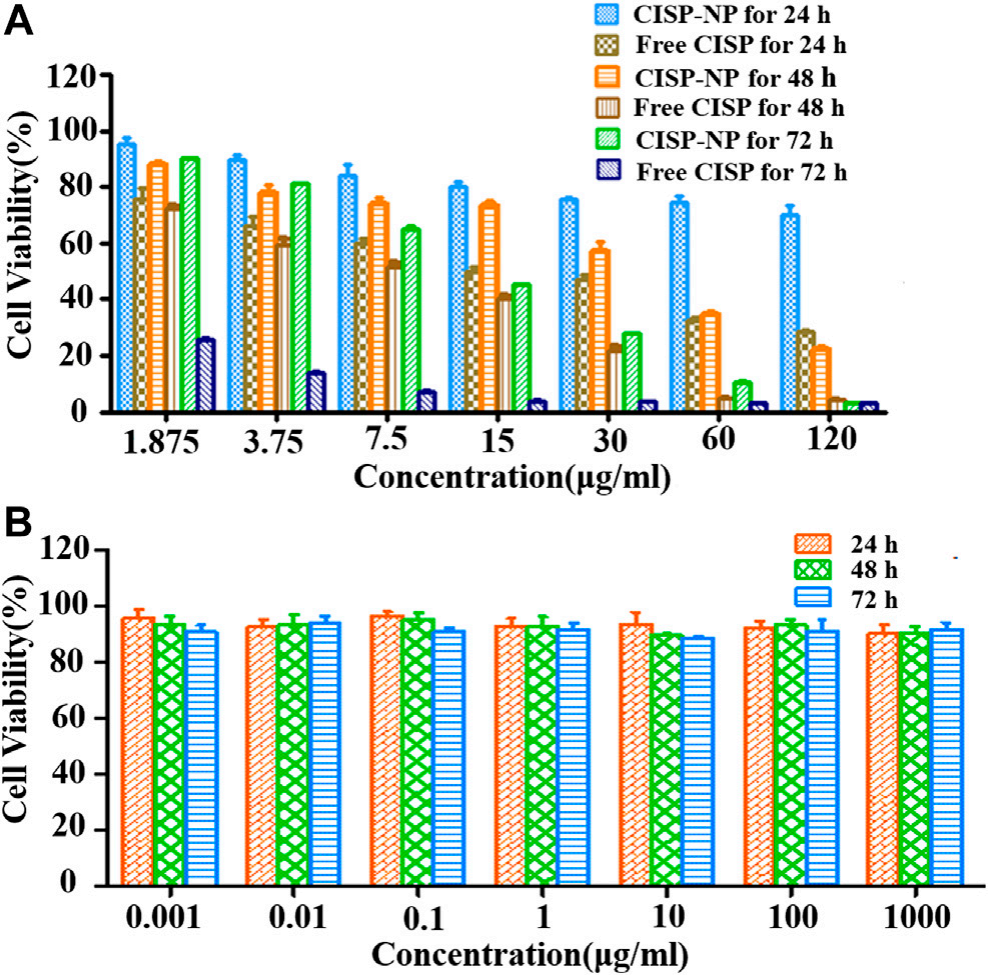
**(A)** Cytotoxicity of free CISP and CISP-NPs at different concentration of CISP on LLC cells for 24, 48, and 72 h. **(B)** Cytotoxicity of blank MPEG_2000_-P(Glu)_1979_ material on LLC cells for 24, 48, and 72 h (n = 6).

To evaluate whether free CISP and CISP-NPs bring into play antineoplastic effect against lung carcinoma, MTT assay was performed to evaluate the cell viability in response to drug treatment in LLC cell lines. Accordingly, both the free CISP and CISP-NPs displayed time-dependent and dose-dependent manner against LLC cells (Figure 7A). Fifty percent growth inhibitory concentration (IC50) was used as the measure of relative cytotoxicity. The IC50 (μg/ml) values of the free CISP group vs. CISP-NPs group were (17.9 vs. 908), (6.789 vs. 33.2), and (3.538 vs. 4.55), at 24, 48, and 72 h, respectively. CISP-NPs showed remarkably lower cytotoxicity than free CISP at 24 and 48 h. CISP-NPs could slightly inhibite the proliferation of LLC cells with cells viability greater than 80%, which were incubated in CISP at a concentration of 15 μg/ml for 24 h. This indicated that most of the Pt complexes came out of the micelle through a ligand exchange reaction and kept active. Then, the conjugation of CISP to the CISP-NPs polymer reduced its *in vitro* cytotoxic activity, but it retained its antitumor activity. However, there was no significant difference between the CISP-NPs group and free CISP group according to the IC50 values at 72 h.

The viabilities of LLC cells treated with MPEG_2000_-P(Glu)_1979_ polymer with concentrations from 0.001 to 1,000 µg/ml after 24, 48, and 72 h incubation were also evaluated (Figure 7B). The blank copolymer micelles were found to be almost nondeleterious, and even if the concentration of the copolymer was up to 1,000 µg/ml, the cell viabilities were still higher than 85.82%. These results indicated that MPEG_2000_-P(Glu)_1979_ polymer was biocompatible with no cytotoxicity and suitably served as a drug delivery vehicle.

### Flow Cytometry Analysis for Cell Cycle

**FIGURE 8:**
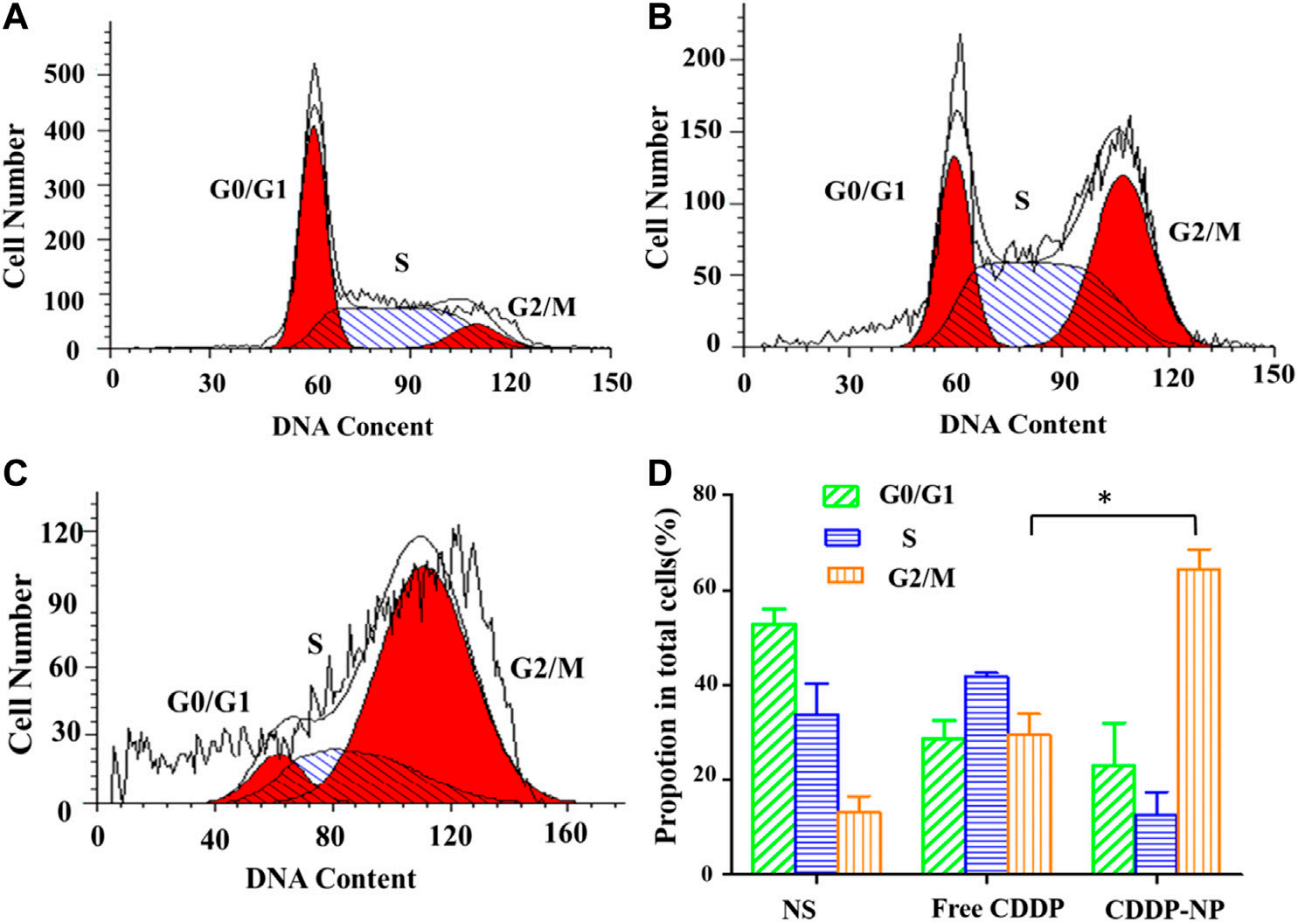
**(A–C)** The cycle kinetics of LLC cells incubated in negative control group (NS) and free CISP, CISP-NPs group at 12.55 μg/ml of CISP for 48 h, respectively. **(D)** Quantitative analysis of cell cycle distribution by flow cytometry of the NS group, free CISP and CISP-NPs group. (n = 3, *p < 0.05).

As shown in Figure 8, after 48 h incubation with different formulations, the results of distribution of LLC cells in the five phases of the cell cycle clearly illustrated that both the group of free CISP and the group of CISP-NPs induced a typical G_2_/M phase arrest in LLC cells. Specifically, the G_2_/M proportions, in quantitative analysis of the cell cycle distribution, were 36.23 and 70.94% for the free CISP group and CISP-NPs group, while the G_2_/M proportion of control group (NS) was only 9.28%. Obviously, LLC tumor cells after CISP-NPs treating displayed a better arrest than the free-CISP-treated group, in terms of the G_2_/M phase. Moreover, cells cycle arrest in the G_0_/G_1_ phase decreased significantly and this would result in enhanced cancer cells death.

### Histopathology Evaluation

**FIGURE 9:**
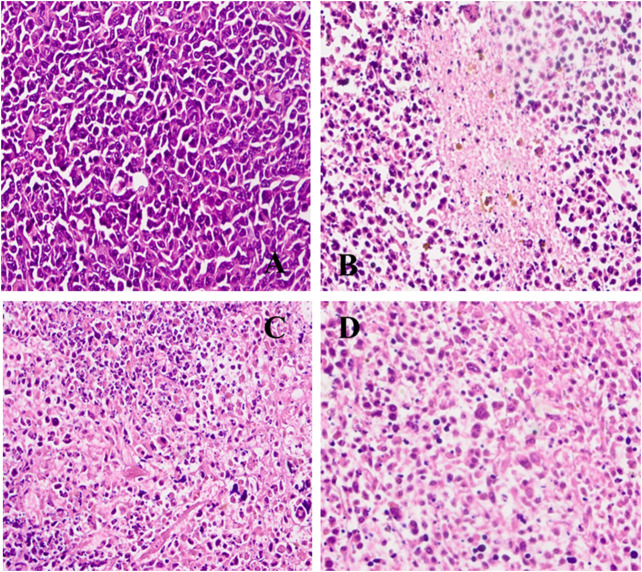
H&E staining of tumor tissue image collected from **(A)** NS group; **(B)** free CISP group; **(C)** CISP-NPs group (6 mg/kg); **(D)** CISP-NPs group (12 mg/kg) (×400).

To further evaluate the safety of CISP-NPs, the analyses of pathology were performed. Cell nucleus was dyed blue by staining with hematoxylin, and, meanwhile, both cytoplasm and extracellular matrix were dyed red by staining with eosin in H&E staining. The cell morphology was not clear, and the chromatin deepened or diffused out of the cell if cell necrosis happened. It was seen that the cells had a large nucleus with a spherical or spindle shape in the tumor tissue of the NS group. At the same time, high nucleocytoplasmic ratio and the soakage of inflammatory cells were seen, which illustrated a fast-growing tumor (Figure 9A). By comparison, in the groups of both CISP-NPs, the tissue is in a state of necrosis to varying degrees, concentrated chromatin was distributed around the edge, and the nuclei of the tumor cells of CISP-NPs (12 mg/kg) group (Figure 9D) were extremely shrinking, fragmented or absent. Of all the tested groups, the CISP-NPs (12 mg/kg) group has the largest necrosis area, whereas CISP-NPs (6 mg/kg) (Figure 9C) administration groups at the same dose levels as free CISP (6 mg/kg) (Figure 9B) showed no significant difference in the degree of necrotic tissue. In addition, PEG could improve the circulation time of the nanoparticles in the bloodstream (Roberts et al., 2012), and CISP-NPs usually had remarkably prolonged blood circulation time in comparison with free CISP.

**FIGURE 10:**
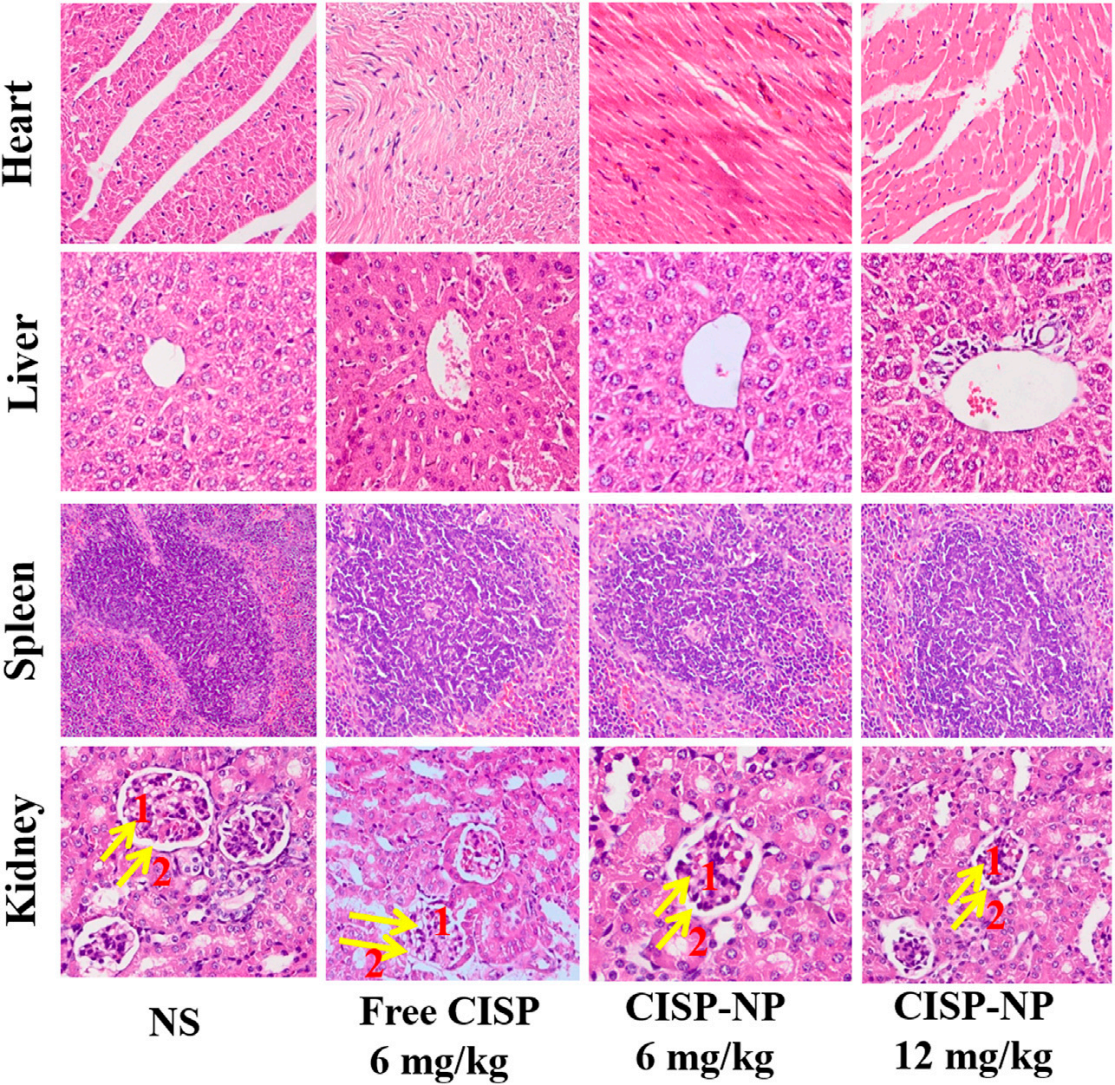
H&E staining of heart, liver, spleen, and kidney tissue excised from LLC tumor-bearing mice following 5 times at 2-day treatment with NS, Free CISP, CISP-NPs (6 mg/kg) and CISP-NPs (12 mg/kg) (yellow arrows one and two represent the glomerular and glomerular wall, respectively, ×400).

As shown in Figure 10, free CISP groups showed obvious damage to kidney, while the groups treated with CISP-NPs were normal. In addition, it was seen that these organs, like heart, liver, spleen, and kidney, had no damage in both CISP-NPs groups. Free CISP disappeared rapidly from circulation and was distributed to each organ. Especially, rapid and high accumulation was observed in the kidney, which was illustrated by the yellow arrows in the figure. NS groups showed a small amount of inflammatory cell infiltration. As for free CISP group (6 mg/kg), tubular epithelial cells in the convoluted proximal tubule became swollen and enlarged. The disappearance of the tubular lumen, cell edema, glomerular wall hyperplasia, nuclear atrophy, and glomerular inflammatory cells infiltration occurred, while CISP-NPs groups (6 mg/kg) were normal. For the CISP-NPs groups (12 mg/kg), due to the larger dose, a small amount of inflammatory cells infiltration was observed, indicating that the CISP-NPs could significantly reduce the nephrotoxicity caused by CISP. Therefore, our results showed that the efficacy of CISP-NPs was statistically equivalent to that of free CISP at the same dosage. However, CISP-NPs have successfully reduced the nephrotoxicity that hampers the clinical application of CISP. Furthermore, the higher concentration of CISP-NPs not only reduced the side effects but also had a better antitumor activity.

The key factors influencing the effect of cellular internalization were the size and composition of particles. PEGylation could enhance the stability and solubility of the drug in plasma and reduce the toxicity of the drug (Saravanan et al., 2017). The renal filtration threshold was about 4–5 nm, and particles with size more than 50 nm were mostly sequestrated by the reticuloendothelial system (RES) of the liver, lungs, kidney, and spleen (Li and Huang, 2008; Dai et al., 2017). The average size of CISP-NPs was (18 ± 1.33) nm, which could efficiently avoid renal clearance for reducing nephrotoxicity and nonselective RES scavenge as well as showing enhanced permeability and retention (EPR) effects of passive targeted drug at solid tumor sites (Figure 3B) (Yang et al., 2014). There are two main factors which contributed to the hypoxia in the tumor microenvironment. The entire tissue was incapable of getting sufficient oxygen due to the abnormal vascular network in the solid tumor, especially for the poorly vascularized internal area. At the same time, the overproliferation of tumor cells further accelerated the consumption of oxygen. Therefore, in the tumor microenvironment, the regions far from the blood vessels were typically hypoxic, which was not conducive to the large size, low penetration of nanoparticles in deep tissues. Jain and coworkers proved that nanoparticles with a 12-nm size had a better tumor penetration than larger sized nanoparticles (Cabral et al., 2011; Chen et al., 2017). To this end, CISP-NPs exhibited significantly superior efficacy in impeding tumor growth with no obvious side effects than that of free CISP in a mouse model of LLC cell line.

## Conclusion

In this study, the CISP-NPs, which was able to reduce side effects obviously and display excellent drug efficacy, were successfully developed. The characterization of CISP-NPs demonstrated that cisplatin was encapsulated in CISP-NPs mostly. Meanwhile, the size distribution of CISP-NPs had a narrow and appropriate distribution, from 8 nm to 40 nm, which contributed to evade renal excretion, prolong systemic circulation, cross tumor blood vessels, and accumulate in tumor tissues due to enhanced permeability and retention effect. The statistics *in vitro* cellular studies revealed that CISP-NPs could release drug slowly so that the duration of action was extended. In addition, MPEG_2000_-P(Glu)_1979_ polymer possessed fine biological compatibility but no systemic cytotoxicity. In further histology evaluation, compared with free cisplatin, CISP-NPs exhibited not only outstanding antitumor activity, but tremendously diminished tissue injury, especially renal toxicity. In conclusion, the present data show that CISP-NPs, reducing nephrotoxicity, are a safe and effective formulation and might be a promising measure to overcome tissue toxicity in antitumor therapy.

## Data Availability

The original contributions presented in the study are included in the article/Supplementary Material; further inquiries can be directed to the corresponding authors.
